# New York Clinics

**Published:** 1913-03

**Authors:** 


New York Clinics. A Bureau of Information has been established at the New York Academy of Medicine, 17-21 West Forty-third street.

